# Supplementary material for: Modelling SARS-CoV-2 Binding Antibody Waning 8 Months after BNT162b2 Vaccination
Source: Vaccines (Basel). 2022 Feb 13;10(2):285. doi: 10.3390/vaccines10020285 (PMC8876471; doi:10.3390/vaccines10020285)
Supplement: Supplementary file 1 [file vaccines-10-00285-s001.zip › vaccines-1568594-supplementary.pdf]

Supplementary Materials

"Modelling SARS-CoV-2 Binding Antibody Waning 8 Months after BNT162B2 Vaccination" – Hatzakis et al.

Table S1. Intervals among two vaccines doses and the four measurements.

**Covariate**	**N = 97**<br>**Median (25th – 75th)**<br>**(days)**
**Interval between first and second dose of COVID-19 vaccine**	21 (21 – 22)
**Interval between first dose of COVID-19 vaccine and**	
first measurement	30 (29 – 32)
second measurement	125 (124 – 126)
third measurement	183 (182 – 185)
fourth measurement	251 (250 – 252)
**Interval between second dose of COVID-19 vaccine and**	
first measurement	9 (8 – 10)
second measurement	103 (102 – 105)
third measurement	161 (160 – 163)
fourth measurement	229 (228 – 231)

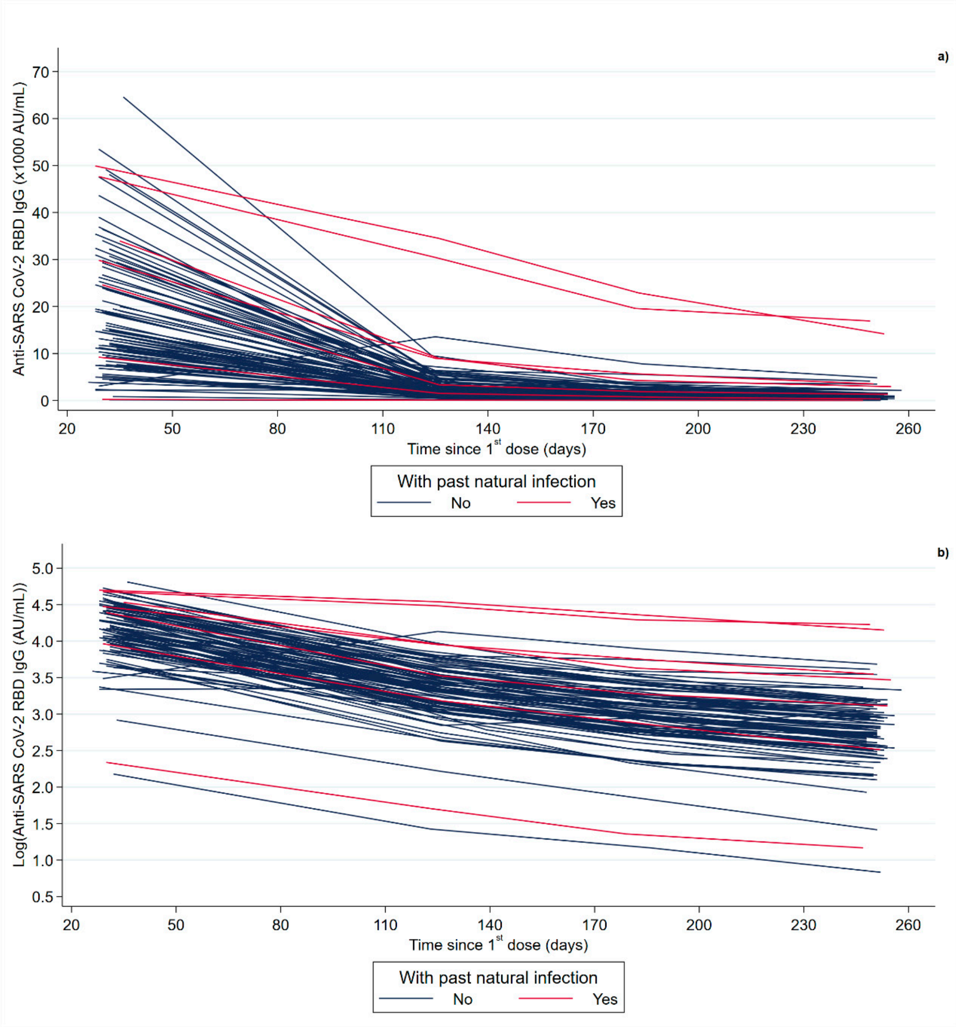

Figure S1. Anti-SARS–CoV–2 RBD IgG antibody levels in **(a)** Normal scale and **(b)** Logarithmic scale, among four-time points per individual. The black color indicates those without past natural infection (N = 90), while the red color indicates those with past natural infection (N = 7).
